# Metabolic reprogramming in cancer: the bridge that connects intracellular stresses and cancer behaviors

**DOI:** 10.1093/nsr/nwaa082

**Published:** 2020-04-30

**Authors:** Yi Zhou, Huiyan Sun, Ying Xu

**Affiliations:** Cancer Systems Biology Center, The China-Japan Union Hospital, China; Department of Biochemistry and Molecular Biology, University of Georgia, USA; Cancer Systems Biology Center, The China-Japan Union Hospital, China; School of Artificial Intelligence, Jilin University, China; Cancer Systems Biology Center, The China-Japan Union Hospital, China; Department of Biochemistry and Molecular Biology, University of Georgia, USA

## Abstract

Extensive changes in cellular metabolisms have been observed in cancer. They are probably induced by the same intracellular stressor, persistent off-balance in intracellular pH across possibly all adult cancers. It is these altered metabolisms that gives rise to a variety of cancerous behaviors such as continuous cell division, metastasis and drug resistance.

Cancer heterogeneity and cancer hallmarks are two characteristics of, most likely, all cancers. The former refers to cancer genomes having diverse changes with little in common at the individual alteration level across different cancer types and different cells in the same tumor [1]; and the latter denotes that all cancers share some complex abnormal behaviors, such as sustained cell proliferation, angiogenesis and metastasis [2]. The coexistence of these two seemingly opposing characteristics is quite puzzling. The first question we address is: what may dictate the large commonalities shared by different cancers?

Different cancers essentially follow the same evolutionary trajectory, consisting of tumor growth, drug resistance, cell migration and metastasis, while the progression rates may differ. As of now, very little has been established regarding the possible reasons that may intrinsically connect these seemingly unrelated behaviors. The second question we examine is: what may drive a cancer to follow essentially the same evolutionary trajectory? We will study these issues from a metabolic and stress-responding perspective.

## REPROGRAMMED METABOLISMS IN CANCER SHARE ONE COMMONALITY

We have previously examined ∼50 known reprogrammed metabolisms (RMs) in 7000+ cancer tissues of 14 cancer types in The Cancer Genome Atlas (TCGA) (Fig. S1 and Table S1), aiming to understand if they may share something in common. Our discoveries are that each of the 50 RMs produces more protons (H^+^) than its original metabolism; and generally the more protons an RM produces, the more up-regulated it tends to be as long as its end-products can be consumed or released [3].

These RMs can be classified into three types: (i) metabolic pathways normally used under specific conditions but persistently up-regulated or repressed throughout a cancer's development; (ii) unusual coupling among normal metabolic processes; and (iii) altered metabolic processes through the utilization of truncated normal metabolisms.

Examples of (i) include persistent up-regulation of *de novo* biosynthesis of nucleotides, biosynthesis of sialic acids, and repression of urea cycle and arginine metabolism. In normal proliferating cells, nucleotides used for DNA syntheses are generated via uptake of nucleosides from circulation and then conversion to nucleotides. In comparison, cancer cells biosynthesize nucleotides using the *de novo* pathway, supplementing it with limited uptake, an observation made decades ago. Further analyses have revealed that cancer cells tend to produce substantially more purine than pyrimidine [4]. Our own research has discovered that the more malignant a cancer is, the more purine it biosynthesizes than pyrimidine [3]. Table 1 summarizes the chemical reactions of the respective biosynthesis processes.

Examples of (ii) are simultaneous synthesis and degradation of triglyceride; simultaneous glycolysis and its reverse process, gluconeogenesis; and choline metabolic cycle. A key observation is that cancers tend to up-regulate both the synthesis and the degradation of a metabolite if both processes produce net protons [3]. For example, the biosynthesis of triglyceride produces one proton per phosphorylation/de-phosphory lation cycle (Fig. S2) and consumes one cytidine triphosphate (CTP), whose over-production in cancer was observed decades ago [5]. And the degradation process produces three protons.

**Table 1.** The overall chemical reactions for *de novo* synthesis of each DNA nucleotide.

purine (dATP)	5-phospho-α-D-ribose-diphosphate + glycine + CO_2_ + 2 10-formyltetrahydrofolate + 2 glutamine + 2 aspartate + 6 ATP + GTP + reduced thioredoxin $ \to $ dATP + 2 tetrahydrofolate + 2 glutamate + 2 fumarate + 6 ADP + GDP + 5 P_i_ + diphosphate + oxidized thioredoxin + **9 H^+^**
purine (dGTP)	ATP + NAD^+^ + reduced thioredoxin + 2 H_2_O $ \to $ dGTP+ 2 tetrahydrofolate + 3 glutamate + fumarate + 6 ADP + AMP + 4 P_i_ + 2 diphosphate + NADH + oxidized thioredoxin + **10 H^+^**
pyrimidine (dCTP)	5-phospho-α-D-ribose-diphosphate + 2 glutamine + aspartate + 6 ATP + FMN + reduced thioredoxin + 2 H_2_O $ \to $ dCTP + 2 glutamate + 6 ADP + 4 P_i_ + diphosphate + FMNH_2_ + oxidized thioredoxin + **5 H^+^**
pyrimidine (dTTP)	5-phospho-α-D-ribose-diphosphate + glutamine + aspartate + 6 ATP + FMN + reduced thioredoxin + 5,10-methylenetetrahydrofolate + H_2_O $ \to $ dTTP + glutamate + 6 ADP + 2 P_i_ + 2 diphosphate + FMNH_2_ + oxidized thioredoxin + 7,8-dihydrofolate + **3 H^+^**

Examples of (iii) include the Warburg effect; truncated pathway of tryptophan degradation; and replacement of the urea cycle by the biosynthesis and release of polyamines for releasing ammonia. There have been numerous proposals regarding the cause of the Warburg effect in the past 90 years. We add one here: it produces one net proton per adenosine triphosphate (ATP) synthesized and utilized. Note that glycolytic ATP generation, shown as is a pH neutral process, while the respiration-based ATP production, written as ${\rm{AD}}{{\rm{P}}^{3 - }}\! +\! {\rm{HP}}{{\rm{O}}_4}^{2 - }\!\rightarrow\!{\rm{AT}}{{\rm{P}}^{4 - }}\! + {\rm{O}}{{\rm{H}}^- }$ consumes one proton for each ATP produced [6]. Hydrolysis of an ATP: ATP^4−^ + H_2_O = ADP^3−^ + HPO_4_^2−^ + H^+^ releases one proton. Hence, glycolytic ATP produces one proton when the ATP is hydrolyzed, while ATP generation by respiration is pH neutral when it is used.


$$\begin{eqnarray*}
{\rm{glucose}} + 2\;{\rm{AD}}{{\rm{P}}^{3 - }} + 2\;{\rm{HP}}{{\rm{O}}_4}^{2 - }\rightarrow \nonumber\\
2\;{\rm{lactat}}{{\rm{e}}^ - } + 2\;{\rm{AT}}{{\rm{P}}^4}^ -
\end{eqnarray*}$$

To understand why cancers alter their metabolisms to produce more protons, we have examined the intracellular pH of the relevant cells. The normal intracellular pH of non-proliferating human cells is slightly acidic at 6.8 and proliferating cells, normal or cancerous, have a slightly alkaline pH at 7.2–7.4 [7]. Normal proliferating cells, such as activated T cells, accomplish this change via increasing the activities of proton exporters and repressing proton importers. Surprisingly, cancer cells do essentially the opposite with increased activities of proton importers and repressed exporters (except for SLC16A1/2) [8]. Taking these together, we hypothesize that there are unknown processes that persistently produce alkaline molecules in cancer cells. Note that the intracellular pH, a fundamental cellular property, must be maintained within a narrow range for the cellular viability.

## FENTON REACTION IN CANCER

It has been established that all cancers are associated with chronic inflammation, hence elevated H_2_O_2_ level. Our literature review has revealed that the majority or possibly all cancers have local iron overload, including colon, lung and breast cancers [9]. The reason for local iron accumulation is due to chronic inflammation beyond a certain level since an oxidative environment can damage the plasma membrane of the local red blood cells (RBCs), leading to their senescence. The senescent RBCs will be engulfed by macrophages and their irons released. In addition, under inflammatory conditions, epithelial cells sequester and hold nearby iron, leading to iron overload over time [10]. Together, they will give rise to cytosolic Fenton reactions, an inorganic reaction without involving enzymes [6]:


$$\begin{equation*}
{\rm{F}}{{\rm{e}}^{2 + }} + {{\rm{H}}_2}{{\rm{O}}_2}\!\rightarrow\! {\rm{F}}{{\rm{e}}^{3 + }} + .{\rm OH} + {\rm{O}}{{\rm{H}}^ - }.\end{equation*}$$

If the environment is also rich in reducing elements such as superoxide $( { \cdot {{\rm{O}}_2}} )$, which is typically the case in an inflammatory environment, this reaction can be rewritten as


$$\begin{equation*}
{\rm{O}}_2^{ \cdot - } + {{\rm{H}}_2}{{\rm{O}}_2} \to \cdot {\rm{OH}} + {\rm{O}}{{\rm{H}}^ - } + {{\rm{O}}_2}\end{equation*}$$

with Fe^2+^ as a catalyst, which will go on so long as the tissue is inflamed and persistently produces **OH^−^** [6]. We have demonstrated that the rates at which OH^−^ is produced in cancer can quickly overwhelm the intracellular pH buffer, hence driving up the pH and killing the host cells if the OH^−^ is not neutralized [6].

Importing protons cannot be a long-term solution since that will change intracellular electric neutrality, a fundamental property that cells must maintain.

Our statistical analysis strongly suggests that all the RMs are induced to neutralize the OH^−^ produced by persistent Fenton reactions to keep the intracellular pH within a livable range [3]. We note that some RMs such as *de novo* nucleotide biosynthesis are highly conserved across all cancers while others are not (Fig. S1), hence each having a distinct metabolic profile and phenotype.

## ALKALOSIS, METABOLIC EXIT AND CANCEROUS BEHAVIOR

We now examine another major stressor encountered by possibly all cancers, finding metabolic exits for some RMs. Normal metabolisms are the result of millions of years of evolution, which has optimized how their products are consumed or released. In comparison, RMs are induced to utilize their ability to produce protons but the host cells may have trouble removing their main or other products, at least for some RMs. Finding novel ways to consume or release such products may give rise to abnormal cellular behaviors. For example, triglyceride synthesis is an RM employed by most cancers (Fig. S1). Some cancers degrade it into glycerol and fatty acids, hence forming a synthesis and degradation cycle, while others partially degrade and release it in the form of arachidonic acid or prostaglandins [3].

### A model for sustained cell division

The most striking example is *de novo* nucleotide biosynthesis, which produces up to 10 protons per nucleotide (Table 1). To be sustained, the synthesized nucleotides must be removed in a timely fashion since otherwise their accumulation will slow down and ultimatel terminate this acidification process based on the law of chemistry. Degradation of such nucleotides is not an option since it is proton-consuming [11]. Their release is not sustainable since they are negatively charged and their release, without a positively charged molecule co-released, will alter the intracellular electric neutrality.

**Figure 1.**
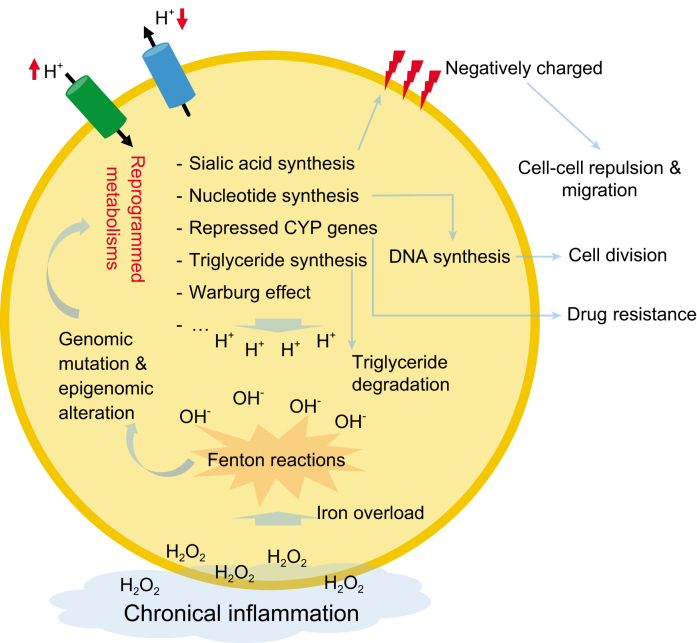
A schematic for our stress-driven cancer development model. Each blue arrow represents ‘leads to’ and the direction of a red arrow indicates up or down regulation.

Our prediction is: cell division serves as a sustainable and potentially most effective way to release nucleotides, coupled with equally but positively charged histones. That is: cancerous cell division is an essential part of a survival process under persistent alkaline stress as otherwise the cells will die from alkalosis.

Interestingly this is how cell division works in unicellular organisms. Such cells take in nutrients and convert them first to energy, ATPs. When the ATP concentration reaches beyond a certain level, the process will slow down and gradually switch to nucleotide biosynthesis as the nutrient uptake continues. The concentrations of nucleotides (or nucleotides-sugar) serve as the cue for the activation of the cell-cycle program [8], switching the cells from a resting state to the proliferating state. We postulate that either a genetic program similar to that in unicellular organisms exists in our genome or an epigenomic program is triggered by stress to help coordinate the complex cell-cycle program. Based on this, we can see that it is the level of Fenton reaction that dictates the rate of nucleotide synthesis, hence the rate of cell division.

### A model for cancer migration driver

Over-production and deployment of sialic acids on cancer cell surface are known to be associated with cancer metastasis since the 1960s but it remains elusive regarding (i) why cancers over-produce sialic acids and (ii) how they contribute to cancer metastasis. We have discovered that the synthesis of each sialic acid produces two protons, and its production correlates with the level of Fenton reaction [3]. In addition, our predicted level of sialic acid accumulation on cell surface strongly correlates with the key characteristics of cell migration such as increased mechanical stress, cell polarity change, cell protrusion and contraction. Based on these and that sialic acid is negatively charged, we have developed a model showing that the gradual accumulation of negatively charged sialic acids on cell surface creates increasingly stronger cell–cell electrostatic repulsion, as well as mechanical stress on, and shape deformation of, the host cells [12]. A previous study has demonstrated that persistent mechanical compression alone, like repulsion here, can drive cell deformation and cell migration in clusters [13]. Hence we predict that it is the persistent production of sialic acids and their gradual accumulation that lead to increasingly stronger repulsion between adjacent cells, driving cancer migration and metastasis [12].

A related issue is: the growth rate and metastasis level of a cancer tends to be complex, and are negatively correlated in some cancers rather than always positively correlated as one might expect [14]. Our model offers a simple explanation to this. The overall relationship between Fenton reaction and all the RMs can be written as follows [3]:


$$\begin{eqnarray*}
{\rm{L}}\left( {{\rm{Fenton}}\;{\rm{reaction}}} \right)\! =\! {{\rm{\alpha }}_1}{\rm{L}}\left( {{\rm{N\ synthesis}}} \right) \nonumber\\
+\, {{\rm{\alpha }}_2}{\rm{L}}\left( {{\rm{SA\ synthesis}}} \right) + \mathop \sum \nolimits {{\rm{\alpha }}_{\rm{i}}}({\rm{R}}{{\rm{M}}_{\rm{i}}}),
\end{eqnarray*}$$

with L(X) denoting the level of X and ${{\rm{\alpha }}_i} \in \mathbb{R}$ being coefficients. Hence when nucleotide and sialic acid syntheses are the predominant acidifiers in a cancer, they will be negatively correlated since their sum is a fixed number, i.e. the level of Fenton reaction. Hence the growth rate (dictated by nucleotide synthesis) and metastasis level (determined by sialic acid synthesis) of such a cancer will be negatively correlated. Otherwise they could be positively correlated or independent as shown in Table S2.

### A model for drug resistance

As a cancer advances it tends to become increasingly drug-resistant, and some cancers are more drug-resistant than others, and yet, their determinants are largely unknown. In drug metabolism, cytochrome P450 enzymes play essential roles and are generally repressed in drugresistant cancers. The reactions catalyzed by such enzymes can be written as [15]:

$$\begin{eqnarray*}
{{\rm{O}}_2} + {\rm{NADPH}} + {{\rm{H}}^ + } + {\rm{RH}} \nonumber\\
\to {\rm{NAD}}{{\rm{P}}^ + } + {{\rm{H}}_2}{\rm{O}} + {\rm{ROH}}
\end{eqnarray*}$$

which consumes one H^+^. We note that generally the higher the Fenton reaction level, the higher the probability that they are repressed. Similar observations are made about the xenobiotic metabolism, the other key player in cancer drug resistance, whose key enzymes tend to be up-regulated because they catalyze proton-producing reactions. Together, they provide an answer, in principle, to the drug-resistance issue.

In conclusion, a common theme throughout this perspective is that it is the stressors, associated with disruption of fundamental homeostasis or encountered when releasing RM products, that drive the cancerous behaviors (Fig. 1). We have found that some other cancerous behaviors such as cachexia could also be explained similarly. Now we can answer the first question raised at the start of this perspective: it is the common stressors such as alkalosis that lead to the same or similar RMs as well as other cancerous behaviors. Regarding the second question: the emergence of new cancerous behaviors such as drug resistance and metastasis as a cancer advances are the result of the natural progression of the increasing stresses or their metabolic responses such as drug resistance as a result of increased intracellular alkalosis and metastasis from the accumulation of sialic acids. Our analyses suggest that RMs provide a rich and potentially powerful framework for cancer research.
